# First Case in Italy of Fatal Intoxication Involving the New Opioid U-47700

**DOI:** 10.3389/fphar.2018.00747

**Published:** 2018-07-10

**Authors:** Enrico Gerace, Alberto Salomone, Clemente Luciano, Daniele Di Corcia, Marco Vincenti

**Affiliations:** ^1^Laboratorio Regionale di Tossicologia, Centro Regionale Antidoping “A. Bertinaria”, Turin, Italy; ^2^Dipartimento di Chimica, Università degli Studi di Torino, Turin, Italy

**Keywords:** U-47700, synthetic opioid, postmortem, intoxication, NPS

## Abstract

The drug commonly known as U-47700 is a strong μ-opioid agonist with an approximate potency 7.5 times higher than morphine. It has been available in Europe since 2014, where it is usually sold through the internet or black market as an abuse morphine-like substance. In the case reported here, a Caucasian man was found dead in his apartment. Next to the body, the police seized one transparent plastic bag containing a white powder and two amber glass bottles with nasal spray containing few milliliters of a transparent liquid. During the autopsy, no evidence of natural disease or trauma was found to account for the death. Blood, urine and pubic hair were collected and submitted for toxicological analysis. The content of the seized materials was also submitted to a general screening analysis in order to determine its composition. U-47700 was detected in blood, urine and hair samples using an UHPLC/MS-MS method purposely developed. The blood and urine concentrations were 380 and 10,400 ng/mL, respectively. No other drugs of abuse nor ethanol were found in blood and urine specimens. Pubic hair analysis revealed a frequent past exposure to U-47700. Finally, U-47700 was identified as the main component of the powder and the liquids contained in the nasal spray bottles. The combined circumstantial elements and toxicological results of the case revealed the occurrence of an acute intoxication produced by U-47700 abuse. To the best of our knowledge, this is the first fatal intoxication case reported on the Italian territory involving the synthetic opioid U-47700.

## Introduction

U-47700 (3,4-dichloro-N-[2-(dimethylamino)cyclohexyl]-N-methylbenzamide) is an opioid analgesic drug developed by the pharmaceutical company Upjohn in the 1970s and structurally related the earlier opioid AH-7921 (Belgian Early Warning System Drug, 2017). At the moment, it is controlled in 3 European countries (Sweden, Finland and United Kingdom) and in the USA. U-47700 is a strong μ-opioid receptor agonist, and reproduces all (or most of the) common effects of opiates such as morphine, including analgesia, pronounced euphoria, sedation and itching (Nikolaou et al., 2017). For these reasons, the compound is gaining popularity on drug user forums as a legal alternative to morphine/heroin (Zawilska, 2017). It is generally sold online as research chemical, not for human consumption, as a white or slightly pinkish powder or fine crystals under different names, including “U4”, “Pink,” or “Pinky.” There is limited information available on the routes of administration and the doses of U-47700 used. It is taken by oral, nasal, rectal routes, or by smoking, intravenous injection, or even by combinations of these routes (Nikolaou et al., 2017; Zawilska, 2017). Side effects, including overdose reactions, are presumed to be very similar to other opiates/opioids as well: depressed respiration (slow breathing), miosis (pinpoint pupils), constipation (World Health Organization, 2016; Baumann and Pasternak, 2018). Even if several cases of acute and lethal intoxication involving U-47700 were reported and reviewed in the literature (Rambaran et al., 2017; Gerace et al., 2018), little is still known about the correlation between its blood concentration and the observed effects. In this investigation, we report a case of fatal intoxication after U-47700 intake. The presence of the drug was confirmed in blood, urine and pubic hair specimens by means of mass spectrometry-based chromatographic methods.

## Background

### Case history

A Caucasian man, with previous history of drug addiction, was found dead in his apartment, lay down on the floor. Two amber glass bottles with nasal spray, containing few mL of a transparent liquid, plus a plastic bag containing a white powder were found on a table near the decedent. Moreover, a package containing a vial of naloxone hydrochloride 0.4 mg/mL was also found on the table. No evidence of violence was observed in the room. The death was reported to the Public Prosecutor who took jurisdiction of the case. To investigate the cause of death, he ordered a post-mortem examination and toxicological analysis.

### Post-mortem examination

The findings were irrelevant, except for a general pulmonary edema. The body appeared well-nourished, and the internal examination presented no evidence of natural disease or trauma to account for his death. At the external examination of the body, no signs of injection was found. To execute the inherent toxicological analyses, heart blood, urine and pubic hair (length: 3 cm) specimens were collected during the post-mortem examination. Peripheral blood was not collected. All of the samples were stored at −20°C before the analysis.

## Experimental

### Samples preparation for fluids and pubic hair

General screening analysis was executed according to a standard procedure employed in our laboratory (Gerace et al., 2014). Briefly, urine sample was extracted with tert-butyl methyl ether (TBME) at alkaline condition after a deconjugation with β-glucuronidase from *E. coli*. After mixing and centrifugation, the organic layer was separated and dried under a nitrogen flow. The residue was reconstituted with 50 μL of methanol and a 1 μL aliquot was injected into the gas chromatography/mass spectrometry (GC/MS) system. In addition, the blood sample was screened with a method for the detection of about ninety pharmaceutical drugs and metabolites routinely employed in our laboratory (Vincenti et al., 2013). For U-47700 quantitation in blood, 50 μL of samples were added with the internal standard (fentanyl-d5) at final concentration of 50 ng/mL and added with 950 μL of acetonitrile/methanol 80:20 (v/v), previously stored at −20°C, and then incubated at −20°C for 15 min. For quantitation in urine 50 μL of samples were added with the internal standard (fentanyl-d5) at final concentration of 50 ng/mL and added with 950 μL of water with formic acid 5 mM/acetonitrile 95:5 (v/v). In both cases, the sample was centrifuged at 14,000 rpm for 5 min and 100 μL of the organic phase was transferred into a new vial. Finally, 1 μL aliquot was directly injected into the UHPLC-MS/MS system operating in selected reaction monitoring (SRM) mode.

Pubic hair analysis was performed on the entire length of the hair lock (3 cm). Approximately 50 mg of hair was twice-washed with dichloromethane and methanol (3 mL each, vortex mixed for 3 min). After complete removal of the solvent wash, the hair was dried at room temperature by a gentle nitrogen flow and subsequently pulverized with a ball mill. For U-47700 quantitation, the hair sample was fortified with 3 μL of a fentanyl-d5 dilute solution used as the internal standard at a final concentration of 0.3 ng/mg. After the addition of 1 mL of methanol, the sample was incubated at 55°C for 15 h without stirring. Finally, the vial was centrifuged once more at 14,000 rpm for 5 min and 1 μL aliquot was directly injected into the UHPLC-MS/MS system operating in SRM mode.

The linear calibration model was checked by analyzing blank hair samples spiked with standard solutions at final concentration of 0, 0.01, 0.025, 0.05, 0.1, and 0.25 ng/mg. Whenever the effective drug concentration exceeded the calibration range, the samples were diluted to fit the quantitation interval considered in the curve.

Moreover, qualitative and quantitative hair analyses for the detection of (i) the most common drugs of abuse, (ii) synthetic cannabinoids and (iii) synthetic cathinones were performed by means of analytical methods used in our laboratory and described elsewhere (Di Corcia et al., 2012; Salomone et al., 2014, 2016).

### Sample preparation for liquids and powder found on the scene

The liquids and the powder found on the scene were subjected to systematic analysis for the detection of drugs and toxic substances. A 100 μL aliquot of liquid and 100 mg of the powder were dissolved in 5 mL of methanol. After sonication in an ultrasound bath for 1 h at 55°C, a 1 μL aliquot of methanolic solution was injected into the GC/MS system with the mass spectrometer acquiring the spectra in the full scan mode (40–650 amu).

### Apparatus and methods

Preliminary screening analyses for amphetamines, tricyclic antidepressants, barbiturates, benzodiazepines, cannabinoids, methadone, cocaine and opiates were performed on urine by the Enzyme Multiplied Immunoassay Technique (EMIT, Abbott Laboratories, IL, USA). The presence of ethanol in the blood was determined by headspace-GC-MS. Screening analysis for unknown substances was performed using a 6890N GC apparatus (Agilent Technologies, Milan, Italy) equipped with a HP−5 17 m fused-silica capillary column (J&W Scientific) with a 0.2-mm inner diameter and a 0.33–μm film thickness. Full scan spectra in the interval 40–650 amu were acquired using a 5,975 inert mass-selective detector (Agilent Technologies, Milan, Italy) operating in the EI mode at 70 eV. The qualitative identification of the underivatized compounds was performed by comparing the full scan spectra obtained with those recorded in the updated spectra libraries (PMWTox2, SWGDRUG version 3.0, AAFS2012, CaymanSpectraLib). For the U-47700 confirmation analysis, a dedicated UHPLC-MS/MS procedure was developed as follows. The chromatographic separation was performed using a Shimadzu LC-30A series system (Shimadzu, Duisburg, Germany) equipped with a CORTECS UPLC C18 column 1.6 μm × 2.1 mm × 100 mm (Waters Corporation, Italy). The elution solvents were water/formic acid 5 mM (solvent A) and acetonitrile/formic acid 5 mM (solvent B). After an initial isocratic condition at 95% A for 0.5 min, the mobile phase composition was varied by a linear gradient (A:B; v/v) from 95:5 to 45:55 in 4.0 min; followed by isocratic elution at 55% B for 0.5 min. The flow rate was 0.5 mL/min and the total run time was 6.0 min including re-equilibration at the initial conditions before each injection. Detection was carried out by an API 5500 triple quadrupole mass spectrometer (ABSCIEX, Foster City, CA, USA) equipped with turbo ion spray source, operating in the positive ionization mode. The SRM transitions used for the determination of U-47700 were 330.9 → 286.1 (quantifier) and 330.9 → 204.1 (qualifier), while for the internal standard the transitions 342.2 → 188.2 was chosen.

### Validation of the LC-MS confirmation methods for U-47700 quantitation

The method was validated by investigating the following parameters: selectivity, linearity, identification and quantitation limits (LOD and LOQ), precision, accuracy and matrix effect. The linear calibration model was checked by analyzing (three replicates) blank samples spiked with U-47700 standard solution at final concentrations of 0, 10, 25, 50, 100, and 250 ng/mL. Whenever the effective drug concentration exceeded the calibration range, the extract was diluted in order to fit the quantitation interval considered in the curve. Ten different blank samples were prepared as previously described to test the selectivity of the whole analytical procedure. The occurrence of possible interferences from endogenous substances was checked by monitoring the signal to noise ratio (S/N) for the U-47700 SRM transitions at the expected retention time. LOD values were estimated as the analyte concentration whose response provided a S/N value equal to 3, as determined from the least abundant transition. The S/N value at the lowest concentration was used to extrapolate the theoretical LOD. This calculated LOD was then experimentally confirmed by analyzing spiked samples at LOD concentration of U-47700. LOQ was calculated as three times the LOD. Within-batch precision (expressed as percent variation coefficient, CV%) and accuracy (expressed as bias %), were assessed by extracting and analyzing a series of ten blood samples fortified at 50 ng/mL. Matrix effect was evaluated by comparing the signal obtained when the analyte was added to the matrix extract with the response obtained from a methanolic solution containing the analyte at the same concentration. The percent difference represented either matrix suppression (value below 100%) or matrix enhancement (value above 100%).

## Results and discussion

The calibration plot showed good linearity in the range 0–250 ng/mL, with a determination coefficient of 0.998. The SRM chromatograms from ten negative samples of blood showed no interfering signals (i.e., S/N ratio lower than 3) at the retention time of U-47700, indicating that the method is selective and free from matrix interferences. The calculated LOD was 0.6 ng/mL and the LOQ was fixed at 2 ng/mL. The results show a satisfactory within-batch precision (CV%: 3.1) and accuracy (bias%: 11.7) at 50 ng/mL. No significant matrix effect was observed (matrix effect 4.0%).

**Figure 1:**
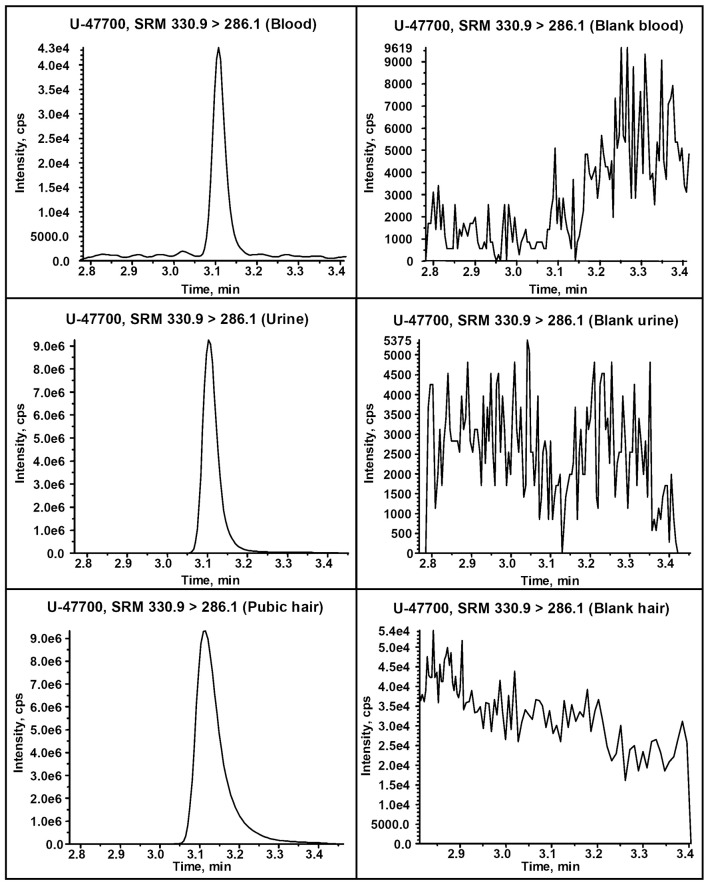
Comparison between the UHPLC-MS/MS extracted ion chromatogram resulting from the detection of U-47700 in the victim's blood, urine and pubic hair (left) and a blank blood, urine and hair samples (right).

The presence of U-47700 was confirmed in all specimens. Figure 1 depicts the SRM profiles obtained from the blood, urine and pubic hair samples for the detection of the target analyte. U-47700 was quantified at a concentration of 380 ng/mL in blood while higher amount of the drug was detected in urine (10,300 ng/mL, creatinine 156 mg/dL). No other drugs nor ethanol were detected in the body fluids. Pubic hair analysis revealed past exposure to U-47700 (5.7 ng/mg). Moreover, pubic hair turned out negative for the presence of traditional drugs of abuse, synthetic cathinones and synthetic cannabinoids. U-47700 was also identified as the main component of the white powder (purity 99%) and of the liquid content of the nasal spray bottles (0.1 mg/ml). The presence of the substance in the nasal spray bottles together with the absence of injection signs on the body indicated that one of the consumption ways was presumably intranasal.

Presently, several intoxication cases related to the consumption of U-47700 alone, or in combination with other drugs, were recently reported (Coopman et al., 2016; Elliott et al., 2016; Mohr et al., 2016; Armenian et al., 2017; Dziadosz et al., 2017; Jones et al., 2017; McIntyre et al., 2017; Papsun et al., 2017; Vo et al., 2017; Koch et al., 2018), but reference toxic and lethal concentrations in body fluids and organs are not yet available for U-47700. Intoxication cases involving U-47700 are summarized in Table 1.

**Table 1:** U-47700 blood concentration in intoxication cases.

**Intoxication outcome**	**Country**	**Year**	**Blood (ng/mL)**	**Specimen^a^ (site of sampling)**	**Other relevant findings (ng/mL)**	**References**
**Fatal**	**Italy**	**2017**	**380**	**cb (heart)**	**None**	**Presented case**
Non-fatal	USA	2016	**7.6**	serum	Fentanyl (15.2), Hydrocodone (107.6), Sertraline (15.7), Gabapentin (350.9)	Armenian et al., 2017
Non-fatal	USA	2016	**228**	serum	None	Jones et al., 2017
Non-fatal	USA	2016	**240**	serum	Phenazepam (1,400)	Vo et al., 2017
Fatal	Germany	2017	**370**	serum	Flubromazepam (830)	Koch et al., 2018
Fatal	USA	2016	**190 340**	pb (femoral) cb (heart)	Alprazolam (120), Doxylamine (300), Diphenhydramine (140), Carboxy-THC (2.4)	McIntyre et al., 2017
Fatal	UK	2016	**1,460**	pb (femoral)	Quetiapine, Amphetamine	Elliott et al., 2016
Fatal	Belgium	2016	**13.8**	pb (subclavian)	Fentanyl (10.9), Sertraline (180)	Coopman et al., 2016
Fatal	Germany	2017	**525 1,347**	pb (femoral) cb (heart)	Diphenidine (1.7), Methoxiphenidine (26)	Dziadosz et al., 2017
Fatal	Germany	2017	**819 1,043**	pb (femoral) cb (heart)	Diphenhydramine (45), Methylphenidate (2.5)	Dziadosz et al., 2017
Fatal	USA	2017	**189**	pb (femoral)	Oxycodone (67)	Papsun et al., 2017
Fatal	USA	2017	**547**	pb (femoral)	Etizolam	Papsun et al., 2017
Fatal	USA	2015–16	**382**	pb (n/a)	Amphetamine (12)	Mohr et al., 2016
Fatal	USA	2015–16	**217**	pb (femoral)	Mephedrone (22), Etizolam	Mohr et al., 2016
Fatal	USA	2015–16	**334**	pb (n/a)	None	Mohr et al., 2016
Fatal	USA	2015–16	**252**	pb (n/a)	Citalopram (43)	Mohr et al., 2016
Fatal	USA	2015–16	**453**	blood	None	Mohr et al., 2016
Fatal	USA	2015–16	**242**	pb (n/a)	Carboxy-THC (5.3)	Mohr et al., 2016
Fatal	USA	2015–16	**103**	n/a	Diphenhydramine (694)	Mohr et al., 2016
Fatal	USA	2015–16	**299**	cb (aorta)	Alprazolam (47), Lorazepam (11), 3-methoxyphencyclidine (180), Tramadol (<250)	Mohr et al., 2016
Fatal	USA	2015–16	**487**	cb (aorta)	Etizolam (86), diphenhydramine (250), chlorpheniramine (<250)	Mohr et al., 2016
Fatal	USA	2015–16	**311**	cb (aorta)	Oxycodone (11), Venlafaxine (2600), o-desmethylvenlafaxine (380)	Mohr et al., 2016
Fatal	USA	2015–16	**59**	cb (aorta)	None	Mohr et al., 2016
Fatal	USA	2015–16	**135**	cb (aorta)	Furanylfentanyl (26), Ethanol	Mohr et al., 2016
Fatal	USA	2015–16	**167**	cb (aorta)	Furanylfentanyl (56), Morphine (48), 6-monoacetylmorphine	Mohr et al., 2016
Fatal	USA	2015–16	**490**	cb (aorta)	Furanylfentanyl (76)	Mohr et al., 2016
Fatal	USA	2015–16	**105**	cb (aorta)	Furanylfentanyl (2.5)	Mohr et al., 2016
Fatal	USA	2015–16	**17**	pb (n/a)	Butyrylfentanyl (26), Ethanol (0.03 g/dL)	Mohr et al., 2016

a *pb, peripheral blood; c, central blood; s, serum; n/a, data not available*.

A brief description of the most significant cases is presented as follows.

A 41-year-old woman presented to the emergency department (ED) for altered mental status, pinpoint pupils and respiratory depression which reversed after 0.4 mg naloxone administration intravenously. The U-47700 concentration in the serum, recorded at the arrival at the ED, was 7.6 ng/mL (Armenian et al., 2017). Other relevant findings included fentanyl (15.2 ng/mL), hydrocodone (107.6 ng/mL), sertraline (15.7 ng/mL) and gabapentin (350.9 ng/mL).

A 26-year-old woman was found cyanotic and with respiratory depression after a nasal insufflation and injection of a product called “U4” (Jones et al., 2017). Toxicological analysis revealed the presence of U-47700 in serum and urine samples at concentrations of 228 and 393 ng/mL, respectively. Also in this case the patient became more responsive after an intravenous naloxone administration.

Another case involving a 29-year-old man found unresponsive after the intravenous injection of U-47700 was described (Vo et al., 2017). The patient regained consciousness spontaneously before the transportation at the ED. Serum sample was positive for U-47700 and phenazepam at a concentrations of 240 ng/mL and 1.4 mg/L, respectively.

A fatal intoxication related to the effect of the U-47700 in combination with the benzodiazepine flubromazepam was recently reported (Koch et al., 2018). A 24 year-old man suffered apnoea and after reanimation and hospital admission, hypoxic cerebral damage and severe brain oedema were stated. Six days after admission mechanical ventilation was discontinued and the patient died. Serum sample collected at the admission to the hospital was positive for U-47700 and flubromazepam at a concentrations of 370 ng/mL and 830 mg/L, respectively.

McIntyre et al. described a fatal intoxication case related to the intake of powder containing U-47700, likely by nasal insufflations (McIntyre et al., 2017). The drug was detected in several post-mortem samples including peripheral blood (190 ng/mL) and the central blood (340 ng/mL). Further presence of U-47700 was determined in liver (1,700 ng/g), vitreous humor (170 ng/mL), urine (360 ng/mL) and gastric content at trace amount (<1 mg).

Another fatal intoxication case associated with the consumption of U-47700 was reported by Elliott et al. (2016). The U-47700 concentration in the femoral blood of a man found dead at home was 1,460 ng/mL.

The presence of U-47700 was found in blood and urine in a case of 30-year old man found dead in his home after inhaling fumes of a powder burned on aluminum foil (Coopman et al., 2016). U-47700 was quantified in post-mortem blood and urine at concentrations of 13.8 and 71 ng/mL, respectively. Toxic levels of fentanyl were also measured in the subclavian blood (10.9 ng/mL). The dead was ascribable to the concomitant intake of U-47700 and fentanyl.

In two cases in which the cause of death was explained by the consumption of U-47700, the concentration of the drug in femoral blood was 525 ng/mL (case 1) and 819 ng/mL (case 2) (Dziadosz et al., 2017). The presence of U-47700 was quantitatively confirmed in additional specimens including heart blood (1,347 ng/mL in case 1 and 1,043 ng/mL in case 2), urine (1,393 and 1,848 ng/mL), kidney (2.7 and 1.4 ng/mg), liver (4.3 and 3.1 ng/mg), lung (3.2 and 2.4 ng/mg), and brain (0.97 and 1.1 ng/mg).

Other two fatal cases involving U-47700 were reported by Papsun et al. (2017). The concentrations of U-47700 in femoral blood were 189 and 547 ng/mL respectively. In the first case oxycodone was also found in blood at significant levels (67 ng/mL) and the death was ascribed to an acute oxycodone and U-47700 overdose. In the second case, the presence etizolam was found together with U-47700. The death was attributed to a U-47700 and etizolam intoxication.

The application of a LC-MS/MS method for the simultaneous analysis of U-47700, U-50488 and furanyl-fentanyl in blood specimens related to 20 postmortem cases, initially attributed to heroin or other opioid-related drug overdoses, was recently described (Mohr et al., 2016). The presence of U-47700 was confirmed in 16 of 20 cases. The U-47700 was the only opioid detected in 9 cases, while in two cases two prescription opioids were detected (tramadol and oxycodone) and in the remaining 5 cases the drug was found in combination with furanylfentanyl (3 cases), morphine and furanylfentanyl (1 case) and fentanyl (1 case). For U-47700, the mean concentration (*N* = 16) was 253 ng/mL, within a range of 17–490 ng/mL.

It is challenging to speculate about which U-47700 levels in blood might be fatal. The available literature deals with cases in which the molecule was detected by means of different analytical techniques in subjects with different characteristics. Furthermore, in most cases U-47700 was not used alone, thus other compounds might have been a contributing factor in the death. In particular, several authors reported the finding of other opioids, including fentanyl and analogs, which likely intensified the central nervous system and respiratory depression. Currently, the sporadic records in which U-47700 was the only detected toxic agent suggest to be cautious before any definitive lethal concentration is presented.

In the present case, the heart blood concentration of U-47700 was comparable with those reported in some other fatal cases previously described, where no other drugs were found to play a role in the intoxication (Mohr et al., 2016). As a matter of fact, the U-47700 blood concentration was also comparable with those recorded in some cases of non-fatal intoxication (Jones et al., 2017; Vo et al., 2017), although in the latter cases these values were obtained from peripheral blood. In one of these cases, there was a prompt resuscitation of the patient at the ED using naloxone (Jones et al., 2017), while in the second case the patient regained consciousness spontaneously (Vo et al., 2017). In the present case, the comparison of the U-47700 concentration in blood and urine (the latter showing a much higher value, also in light of the high density) suggests the occurrence of extensive drug excretion and possibly a long agony before death. Moreover, the presence of U-47700 in the pubic hair sample indicates that the decedent had previously been exposed to the same drug on more than one occasion.

## Concluding remarks

In the fatal case reported here, the intake of U-47700 was proved and suggests that its depressant effect on the central nervous system was likely to account for the consumer's death. U-47700 was present in body fluids at a concentration compatible with acute intoxication conditions, possibly leading to death. To the best of our knowledge, this is the first reported case of fatal intoxication involving U-47700 that occurred on the Italian territory. Intoxication cases involving NPS, including U-47700, continue to pose challenges for toxicologists. Deaths and intoxication cases consistent with opioid consumption, but negative to the traditional drug screenings, should be subjected to further testing for the detection of fentanyl analogs and novel opioid-like compounds. Moreover, in all fatalities involving opiates and opioids, where the toxic effect is related with acquired tolerance through frequent use, the interpretation of post-mortem drug concentration may be challenging. The absence of reference concentrations in post-mortem matrices together with the contribution of other drugs to the intoxication makes the interpretation even more problematic.

## Informed consent for publication

This manuscript does not violate the privacy of the deceased, nor contain identifiable details and therefore the anonymity is maintained. The Institution represented by AS was informed and consequently waived the request for informed consent from the next of kin of the deceased.

## Author contributions

All authors had full access to all of the data in the study and take responsibility for the integrity of the data and the accuracy of the data analysis. EG and AS: case study concept and design. EG, AS, CL, and DD: acquisition, analysis, or interpretation of data; EG, AS, and MV: drafting of the manuscript; All authors: critical revision of the manuscript for important intellectual content; All authors: study supervision.

### Conflict of interest statement

The authors declare that the research was conducted in the absence of any commercial or financial relationships that could be construed as a potential conflict of interest.
